# A strategy for the identification of combinatorial bioactive compounds contributing to the holistic effect of herbal medicines

**DOI:** 10.1038/srep12361

**Published:** 2015-07-22

**Authors:** Fang Long, Hua Yang, Yanmin Xu, Haiping Hao, Ping Li

**Affiliations:** 1 State Key Laboratory of Natural Medicines, China Pharmaceutical University, Nanjing 210009, China

## Abstract

It has been well claimed that herbal medicines (HMs) elicit effects via a multi-compounds and multi-targets synergistic mode. However, it lacks appropriate strategies to uncover the combinatory compounds that take effect together and contribute to a certain pharmacological effect of an herb as a whole, which represents a major bottleneck in providing sound evidence in supporting the clinic benefits of HMs. Here, we proposed a strategy to the identification of combinatory compounds contributing to the anti-inflammatory activity of Cardiotonic Pill (CP). The strategy proposed herein contains four core steps, including the identification of bioequivalent combinatorial compounds, chemical family classification-based combinatorial screen, interactive mode evaluation, and activity contribution index assay. Using this strategy, we have successfully identified six compounds in combination responsible for the anti-inflammatory effect of CP, whose anti-inflammatory activities were found comparable to that of the whole CP. Additionally, these six compounds take effect via an additive mode but little synergism. This study, together with our recent work in the identification of bioactive equivalent compounds combination, provides a widely applicable strategy to the identification of combinatory compounds responsible for a certain pharmacological activity of HMs.

With the understanding that the pathogenesis of many diseases involves multiple factors, the focus of drug discovery has shifted from the conventional “one target, one drug” model to a new “multi-target, multidrug” model^1^,^2^,^3^,^4^. Plants are excellent sources of bioactive compounds throughout history in the search for new drugs^5^,^6^,^7^,^8^,^9^,^10^. Moreover, it has been well claimed that herbal medicines (HMs) are by themselves multi-component mixtures and elicit effects via a multi-targets additive and/or synergistic mode^11^,^12^,^13^,^14^,^15^,^16^ and thus may fit well for the requirment in the therapy of multi-gene related complex diseases. Indeed, accumulating evidence from clinical studies support that HMs represent an efficient form of therapy in the control of complex diseases, such as cardiovascular disease (CVD), cancer and diabetes^17^. The FDA has approved the clinical trials of more and more herbal medicines in the past years, which represents a positive attitude of Western countries in the evaluation of HMs. Furthermore, HMs have been claimed as unique natural templates in the *de novo* discovery and development of multi-compounds and multi-targets innovative drugs^18^,^19^. On both cases, however, a basic requirement is to uncover the pharmacologically active compounds in combination that can represent the holistic clinical benefits of the whole HMs. Previous efforts to the identification of bioactive compounds have focused on the screening of isolated and single bioactive compound from HMs and made great contributions to the discovery and development of new drugs. However, such screening strategies are hard to uncover the combinatory compounds contributing to the holistic effect of HMs, which represents a major bottleneck in providing sound evidence in supporting the clinic benefits of HMs^20^,^21^.

More recently, our laboratory has proposed a strategy called “bioactive equivalent combinatorial components (BECCs)” to uncover pharmacologically active compounds in combination that are representative of the holistic effect of the whole HMs^14^. BECCs is defined as the exact composition of combinatorial components accounting for the whole efficacy of original herbal medicines. Using this strategy, we have successfully discovered a combination of 18 compounds (Supplementary Figure S1) as BECCs of Cardiotonic Pill (CP), which has been used for the therapy of CVD for decades of years in China and has recently been approved to enter Phase III clinical trials by the FDA^22^,^23^,^24^. Many pharmacological activities of CP or the compounds contained have been reported in supporting its clinical therapeutic effect towards CVD, which mainly include anti-inflammatory, scavenging free radical, improving microcirculatory, lipid-lowering, vasodilatory, anti-coagulant, anti-thrombotic, anti-ischemia, anti-apoptotic, endothelium-protective, and mitochondria-protective effects^25^,^26^,^27^,^28^,^29^. However, it still remains a critical question of what compounds in combination, acting in a synergistic and/or additive mode, contribute to what pharmacological activities, which is a key step to finally uncovering the multiple-compounds and multiple-targets holistic mode of HMs.

Systems biology has revealed a complex array of pathological processes underlying CVD, such as inflammation, oxidative stress, accumulation of lipids, coagulation, endothelial cell injury, ischemic injury, apoptosis and mitochondrial dysfunction^30^,^31^,^32^. Among all of these pathological processes and causes, inflammation represents a core throughout the whole process of pathological development of CVD^33^,^34^,^35^,^36^,^37^. The activation of inflammatory cells evokes the release of inflammatory cytokines, chemokines, oxygen and nitrogen radicals, and other inflammatory molecules, ultimately, the overactive inflammatory response leads to the injury of heart muscle and cause both structural and functional deficits. Therefore, timely repression of the inflammatory response is critical for effective healing of the injured tissues. Many experimental and clinical investigations have reported that CP can suppress the inflammatory responses and show a cardio-protective capacity^38^,^39^,^40^,^41^,^42^.

As an extension of our recent study, the current study aims to develop and validate a strategy to the identification of combinatorial compounds which take effect together contributing to a certain pharmacological benefit of HMs, using the anti-inflammatory activity of CP as a typical model. This strategy mainly includes the following four steps (Fig. 1): (1) the identification of BECCs via a bioactive equivalence oriented feedback screening approach; (2) screening active compounds based on chemical family classification; (3) determining the pharmacological activity of individual compounds and their interactive mode; (4) designation of dominant compounds combination based on activity contribution assay, which is followed with a validation of activity.

## Results

### Characterization of anti-inflammatory activities in lipopolysaccharide (LPS)-stimulated macrophages

**Figure 2:**
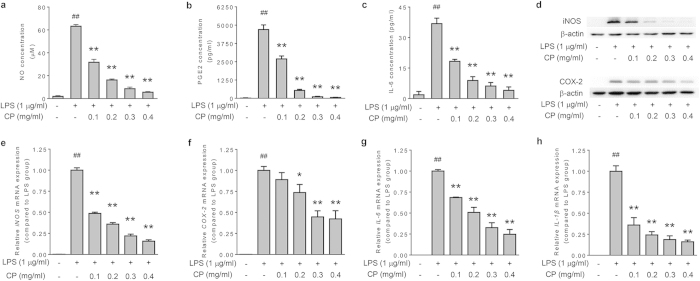
Anti-inflammatory effects of Cardiotonic Pill in LPS-stimulated RAW264.7 macrophages. Cells were pretreated with different concentrations of CP (0.1–0.4 mg/ml) for 1 h, followed by treatment with LPS (1 μg/ml) and incubation for indicated time. Concentration of (**a**) NO, (**b**) PGE_2_ and (**c**) IL-6 in the supernatant. (**d**) Protein expression levels of iNOS and COX-2. β-actin was used as an internal loading control. The expression levels of mRNA for (**e**) iNOS, (**f**) COX-2, (**g**) IL-6 and (**h**) IL-1β were analyzed by quantitative real-time PCR. GAPDH served as internal control for normalization of mRNA expression. Data are presented as mean ± SD of three independent experiments performed in duplicate. ##p < 0.01 versus control group; *p < 0.05, **p < 0.01 versus LPS group (one-way ANOVA, Dunnett test). CP: Cardiotonic Pill.

To investigate the anti-inflammatory effects of CP, we stimulated the murine macrophage cell line RAW264.7 with LPS, treated cells using various concentrations of CP and measured the production of inflammatory mediators. CP significantly inhibited the LPS-induced production of nitric oxide (NO), prostaglandin E_2_ (PGE_2_) and interleukin-6 (IL-6) in a dose-dependent manner (Fig. 2a–c, p < 0.01). The protein and mRNA expression levels of inducible nitric oxide synthase (iNOS) and cyclooxygenase-2 (COX-2) were also reduced by pre-incubation of cells with CP (Fig. 2d–f). Additionally, CP concentration-dependently decreased the expression of IL-6 and interleukin-1β (IL-1β) mRNA (Fig. 2g,h). To ensure that cell death was not responsible for the decreased cytokine expression in CP-treated group, the cytotoxicity of CP was evaluated in the presence of LPS. Incubation with different concentrations of CP (0.1, 0.2, 0.3, 0.4 and 0.5 mg/ml) for 24 h did not cause any significant viability changes compared to the control group (Supplementary Figure S2). These data suggest that CP treatment can suppress the expression of inflammatory mediators and reduce the inflammatory response induced by LPS. Because CP at 0.4 mg/ml showed strong anti-inflammatory activities and had little toxic effect against macrophages, this concentration was applied in the further studies for the identification of combinatorial compounds explaining for the anti-inflammatory activities of CP as a whole.

### Chemical family classification-based screening

**Figure 3:**
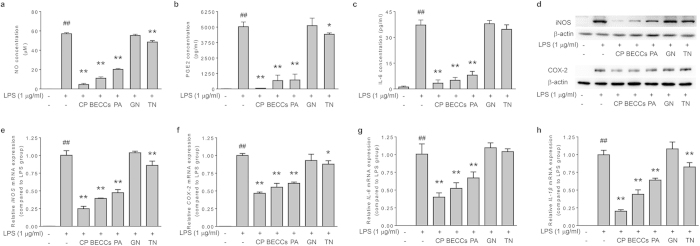
Chemical family classification-based screening of anti-inflammatory compounds from CP. Cells were pretreated with tested samples (at a corresponding concentration in 0.4 mg/ml CP) for 1 h, followed by treatment with LPS (1 μg/ml) and incubation for indicated time. Concentration of (**a**) NO, (**b**) PGE_2_ and (**c**) IL-6 in the supernatant. (**d**) Protein expression levels of iNOS and COX-2. β-actin was used as an internal loading control. The expression levels of mRNA for (**e**) iNOS, (**f**) COX-2, (**g**) IL-6 and (**h**) IL-1β were analyzed by quantitative real-time PCR. GAPDH served as internal control for normalization of mRNA expression. Data are presented as mean ± SD of three independent experiments performed in duplicate. ##p < 0.01 versus control group; *p < 0.05, **p < 0.01 versus LPS group (one-way ANOVA, Dunnett test). CP: Cardiotonic Pill; PA: combination of 10 phenolic acids; GN: combination of 4 ginsenosides; TN: combination of 4 tanshinones; BECCs: bioactive equivalent combinatorial components (combination of PA, GN and TN).

Using a bioactive equivalence oriented feedback screening strategy, a combination of 18 compounds has been identified as BECCs of CP in our previous work^14^. We thus decided to identify the compounds in combination responsible for the anti-inflammatory activities of CP from the previously identified BECCs. To this end, we firstly validated whether or not the BECCs is bioactive equivalent to CP in anti-inflammation by using the model of LPS-stimulated RAW264.7 cells. Pretreatment with BECCs significantly reduced the production of NO, PGE_2_ and IL-6 (Fig. 3a–c, p < 0.01). BECCs also repressed the expression of iNOS and COX-2 at the mRNA and the protein level (Fig. 3d–f). The mRNA levels of IL-6 and IL-1β were also decreased upon BECCs treatment (Fig. 3g,h). Consistent with our previous result, we validated in this study that BECCs could explain a large part, if not all, of the anti-inflammatory effect of CP extract. These results support that it is reasonable to identify the combinatory compounds with anti-inflammatory activities from this set of 18 compounds.

It has been widely acknowledged that the compounds contained in HMs can usually be classified into several chemical families; moreover, a family of chemical compounds characterized with similar pharmacophores may possess similar pharmacological activities, despite of different intensities. From this consideration, we proposed an approach of chemical family classification-based screening to facilitate the process of the identification of compounds with anti-inflammatory activities. The 18 compound in CP can be classified into three families on the basis of their chemical structures, including phenolic acids (PA), ginsenosides (GN), and tanshinones (TN) (Supplementary Figure S1). To identify which group of constituents is responsible for the anti-inflammatory activity of CP, the effects of PA, GN and TN on the production of NO, PGE_2_ and IL-6 were determined. We prepared PA, GN, TN, and their combination (BECCs) by mixing reference compounds according to the corresponding concentration of each compound in 0.4 mg/ml of CP (Supplementary Table S1). PA significantly inhibited the production of NO, PGE_2_ and IL-6 (Fig. 3a–c, p < 0.01), and decreased the protein levels of iNOS and COX-2 in LPS-stimulated RAW264.7 cells (Fig. 3d). TN also presented certain inhibitory effects in LPS-stimulated inflammatory mediator production. The analysis of mRNA of iNOS, COX-2, IL-6 and IL-1β suggested a similar tendency (Fig. 3e–h). The results indicate that PA and TN, but not GN, are primary responsible for the anti-inflammatory effects of CP.

### Screening single compounds and characterizing interactive mode

**Figure 4:**
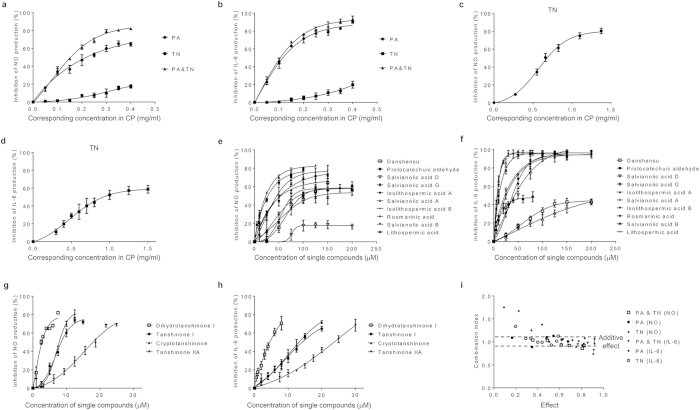
Combination index and dose-dependent inhibition of LPS-induced inflammatory indicator production by single active compounds or compound combinations. RAW264.7 macrophage cells were pretreated with different concentrations of tested samples in the presence of 1 μg/ml LPS for 24 h. Dose-effect curves of PA, TN, and their combinations in inhibition of (**a**) NO and (**b**) IL-6 production. Dose-effect curves of TN at higher concentrations in inhibition of (**c**) NO and (**d**) IL-6 production. Dose-effect curves of 10 phenolic acids in inhibition of (**e**) NO and (**f**) IL-6 production. Dose-effect curves of 4 tanshinones in inhibition of (**g**) NO and (**h**) IL-6 production. (**i**) Combination index. The combination index was calculated using CompuSyn software based on the data of 3 replicates. 0.9 < CI < 1.1 indicated an additive effect. CP: Cardiotonic Pill; PA: combination of 10 phenolic acids; TN: combination of 4 tanshinones; PA&TN: combination of PA and TN.

Production of NO and IL-6 in LPS-stimulated RAW264.7 cells was employed to screen the single compounds with anti-inflammatory activity and to evaluate the interactive mode. The abilities for suppressing inflammatory indicator levels in LPS-stimulated RAW 264.7 cells of single compounds, combinations of 10 phenolic acids (PA), combinations of 4 tanshinones (TN), combinations of PA and TN were tested at various concentrations (Fig. 4). RAW264.7 cell viability was measured to ensure that cell death was not responsible for the decreased production of inflammatory indicator (Supplementary Figure S3). IC_50_ values of single compounds in PA and TN are listed in Table 1. PA and TN, either alone or in combinations, were able to suppress the accumulation of NO and IL-6 in a concentration-dependent manner (Fig. 4a–d). The combination of PA and TN inhibited 50% of NO and IL-6 production at a corresponding concentration in CP of 0.15 mg/ml and 0.10 mg/ml, respectively. A combination index (CI) method was used for assessing the nature of the interaction (synergistic, additive, or antagonistic effect)^43^,^44^,^45^,^46^,^47^. CI is calculated on the basis of drug concentration and biological activity (Supplementary Table S2–S4), and the plotting of dose-effect curves is a prerequisite step for evaluation of combination effects. As showed in Fig. 4i, most of the CI values located in the range of 0.9 to 1.1, indicating an additive effect between PA and TN. In addition, interactive mode among the compounds in PA and TN were also determined. The data show that the interactions among the compounds in PA family, as well as the compounds in TN family, were also mainly in an additive mode (Fig. 4i).

### Contribution coefficient and dominant compounds combination

To elucidate the holistic effect of HMs, it is important to determine the contribution of each compound and the interactive mode. Results obtained from the study above indicate an additive mode among the compounds contained in CP in anti-inflammatory response. We thus tuned to determine the contribution of each compound to the whole anti-inflammatory effects of CP. In the absence of synergism or antagonism, the combined effect can be predicted by the mass-action law principle^48^,^49^. Based on the additive effect of active compounds in this study, we were able to determine the contribution coefficient of each compound to whole anti-inflammatory effect of CP. Activity contribution index at the median effect (ACI_50_) was used to evaluate the activity contribution. Table 1 shows the ACI_50_ of each compound in the combination of PA and TN that contributes to the inhibition of 50% of NO and IL-6 production.

According to the ACI_50_ of each compound, we select the top major contributors, for which the sum of ACI_50_ exceeds 85%, as the dominant combination of anti-inflammatory compounds (DCAC) of CP. Using NO production as the indicator, protocatechuic aldehyde, danshensu, dihydrotanshinone I, salvianolic acid A, isolithospermic acid A, and tanshinone I were identified as the top six compounds contributing to the anti-inflammatory activity of CP. The sum of the ACI_50_ of these six compounds is 85.6%. Considering that the top contributors may vary from different pharmacological indicators applied, we used IL-6 production as another indicator to validate whether the six compounds determined were representative of the anti-inflammatory activities of CP. Although the rank of ACI_50_ varied from that of NO production, the sum of ACI_50_ values of the six contributors was 84.7% for the inhibition of IL-6 production. These results suggest that the combination of these six contributors could be representative of the whole anti-inflammatory activity, and they were thus designated as the dominant combination of anti-inflammatory compounds (DCAC) of CP.

### Anti-inflammatory effect validation of DCAC

**Figure 5:**
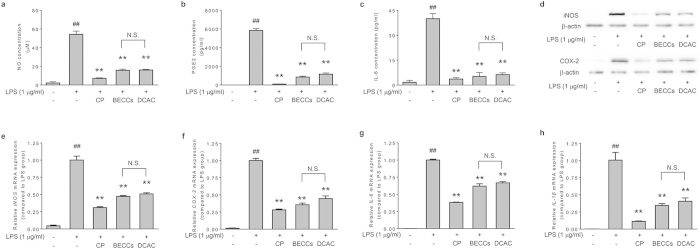
Anti-inflammatory effect validation of DCAC in LPS-stimulated RAW264.7 macrophages. Cells were pretreated with tested samples (at a corresponding concentration in 0.4 mg/ml CP) for 1 h, followed by treatment with LPS (1 μg/ml) and incubation for indicated time. Concentration of (**a**) NO, (**b**) PGE_2_ and (**c**) IL-6 in the supernatant. (**d**) Protein expression levels of iNOS and COX-2. β-actin was used as an internal loading control. The expression levels of mRNA for (**e**) iNOS, (**f**) COX-2, (**g**) IL-6 and (**h**) IL-1β were analyzed by quantitative real-time PCR. GAPDH served as internal control for normalization of mRNA expression. Data are presented as mean ± SD of three independent experiments performed in duplicate. ##p < 0.01 versus control group; *p < 0.05, **p < 0.01 versus LPS group (one-way ANOVA, Dunnett test). CP: Cardiotonic Pill; BECCs: bioactive equivalent combinatorial components; DCAC: dominant combination of anti-inflammatory compounds.

To further evaluate whether or not DCAC, a set of six selected compounds, could be representative of the whole anti-inflammatory activity of CP, the anti-inflammatory effects of DCAC was validated in LPS-stimulated RAW264.7 cells in comparison with BECCs and CP extract. DCAC treatment significantly reduced the production of NO, PGE_2_ and IL-6 (Fig. 5a–c, p < 0.01). Western blot analysis showed that the protein expression of iNOS and COX-2 in RAW264.7 cells was also suppressed by the pretreatment of DCAC (Fig. 5d). Likewise, DCAC treatment remarkably decreased the mRNA levels of iNOS, COX-2, IL-6 and IL-1β (Fig. 5e–h). Notably, the anti-inflammatory effects of DCAC were found comparable to that by BECCs, in terms of all the parameters determined. These results support that the designated DCAC is dominantly responsible for the anti-inflammatory effects of CP.

**Figure 6:**
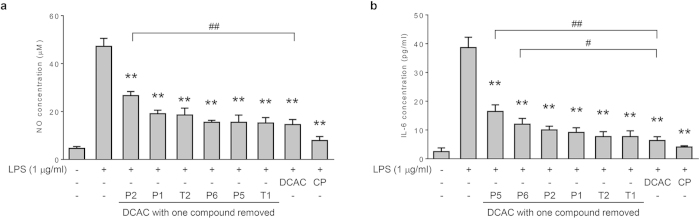
Effects of compound removal on the anti-inflammatory activity of DCAC. Cells were pretreated with tested samples (at a corresponding concentration in 0.4 mg/ml CP) for 1 h, followed by treatment with LPS (1 μg/ml) and incubation for 24 h. (**a**) NO and (**b**) IL-6 concentration in the supernatants of macrophages treated by DCAC with each compound removed respectively. Data are presented as mean ± SD of three independent experiments. **p < 0.01 versus LPS group; #p < 0.05, ##p < 0.01 versus DCAC group (one-way ANOVA, Dunnett test). CP: Cardiotonic Pill; DCAC: dominant combination of anti-inflammatory compounds; P1: danshensu, P2: protocatechuic aldehyde; P5: isolithospermic acid A; P6: salvianolic acid A; T1: tanshinone I; T2: dihydrotanshinone I.

To further validate that the ACI-based selection of top compounds was appropriate, we removed the compound one by one from the six compounds and the anti-inflammatory activity of the remaining five compounds was tested (Fig. 6). When protocatechuic aldehyde, which is the leading activity contributor for inhibition of NO production (ACI_50_ 33.4%), was removed from DCAC, the inhibitory activity of the remaining combination was significantly reduced (Fig. 6a, p < 0.01 compared to DCAC). In contrast, when tanshinone I (ACI_50_ 4.8%) was removed, the activity of the remaining part was not significantly affected. The analysis of IL-6 production suggested a similar tendency (Fig. 6b). When isolithospermic acid A (ACI_50_ 37.1%) was removed from DCAC, the inhibitory activity of the remaining combination in inhibition of IL-6 production was significantly reduced (Fig. 6b, p < 0.01 compared to DCAC). These results strongly indicate that ACI-based selection is an appropriate strategy to the identification of combinatorial compounds from HMs.

## Discussion

In recent years, it has been increasingly acknowledged that HMs are by themselves multi-component mixtures with a ‘fixed dose’ taking effect via an additive and/or synergistic mode and thereby manifest a holistic clinic benefits^12^,^15^,^16^. Such an additive and/or synergistic mode may explain why the original HMs usually has a superior activity over the isolated single constituents. The identification of bioactive compounds in combination that take effect in an additive and/or synergistic mode from HMs is thus of great importance, not only for providing scientific evidence for the clinic benefits of HMs, but also for the quality control and the development of new drugs^21^,^50^. However, the lack of appropriate strategies and methodologies represents a major hindrance in addressing this challenging and important task. On the basis of our recent identification of a set of 18 compounds which explain the majority of the holistic effects of CP, this study contributes to the development of a combinatory screening strategy and the identification of six compounds in combination responsible for the anti-inflammatory effect of CP. The anti-inflammatory activities of these six compounds in combination were found comparable to the whole extract of CP. Moreover, we further showed that these six compounds take effect via an additive mode but little synergism.

Theoretically, HMs may elicit the holistic effects via multiple compounds targeting various targets and thus fitting for the requirement of combating diverse pathological factors of complex and multi-gene diseases^12^,^15^,^16^,^19^. Additionally, it is also important to note that the level of single compound contained in HMs is usually too low to exert sufficient pharmacological activity against certain pathological processes or factors. Therefore, it is probably that several compounds in combination acting via a synergistic and/or additive mode combating against the same pathological processes, via targeting to either the same or diverse targets. From this consideration, this study focused on the evaluation of such a holistic mode of CP in anti-inflammatory activity, which is believed to be a major mechanism in explaining the clinical benefits of CP to cardiovascular diseases. First, we have validated that the previously identified BECCs, a set of 18 compounds in combination, was comparable with the CP extract in terms of anti-inflammatory effects. Second, we proposed a chemical family classification-based screening strategy to rapidly identify what family of compounds is responsible for the anti-inflammatory effects of CP. For this purpose, we prepared the combinations of three family of compounds, PA, TN, and GN, by mixing the standards of each compound at their original concentration in CP extract. It was found that PA and TN, but not GN, exert strong anti-inflammatory activity. Thereafter, compounds of GN family were thus excluded from further screening. This result supports that the chemical family classification-based screening approach is very feasible and efficient to narrow the screening scope. Third, the anti-inflammatory activity of compounds in PA and TN family was individually screened and the IC_50_ value of each compound was determined. Moreover, we extended to determine the interactive mode between two families of compounds and among the compounds in the same family. Of interest, the results showed that these compounds in combination take effect in an additive mode. On the basis of this finding, we utilized the mass-action law principle to predict the contribution index of each compound to the whole anti-inflammatory activity of CP. With this result, six compounds including protocatechuic aldehyde, danshensu, dihydrotanshinone I, salvianolic acid A, isolithospermic acid A and tanshinone I were designated as the dominant combinatory compounds of CP in terms of anti-inflammation efficacy. Although the anti-inflammatory activities of such compounds have been previously reported^44^,^45^,^46^,^47^,^48^, the current study contributes, for the first time, to the elucidation of the exact of contribution of each compound to the whole anti-inflammatory activity of CP. Moreover, we found some other compounds with well known anti-inflammatory activities have little contribution to the holistic effect of CP because of their very low level in CP. For example, cryptotanshinone possesses a strong anti-inflammatory activity characterized with IC_50_ value at 8.0 μM but was not included into DCAC because of its low level in CP extract. Unlike the screening for individual bioactive compounds from HMs, the current study focuses on the elucidation of which compounds take effect in a combination and contribute to the holistic effect, and thereby provides evidence in explanation of the clinical benefits of HMs.

In addition to the anti-inflammatory activity, CP possesses many other pharmacological effects such as anti-oxidant and metabolic regulation, all of which may contribute to the observed clinical benefits of CP. Therefore, the current study is far from conclusive in terms of the elucidation of the multi-compounds and multi-targets mode of CP against cardiovascular diseases. The search for the combinatory compounds contributing to other pharmacological effects of CP is now ongoing in our laboratory. In conclusion, this study, together with our recent work, provides a widely applicable strategy to the identification of combinatory compounds responsible for a certain pharmacological activity of HMs. The dominant compounds combination obtained from this strategy not only provides insights to the understanding of the holistic effects, but may also shed light on the selection of appropriate maker compounds for clinical benefits associated quality control of HMs.

## Methods

### Reagents and chemicals

Chemical standards for danshensu, protocatechuic aldehyde, salvianolic acid A, salvianolic acid B, salvianolic acid D rosmarinic acid, lithospermic acid, dihydrotanshinone I, cryptotanshinone, tanshinone I, and tanshinone IIA were purchased from Chengdu Must Bio-Tech Co. Ltd. (China). Ginsenosides Rg1, Rb1, Rh1, and Rd were purchased from Jilin University (China). Isolithospermic acid A, isolithospermic acid B and salvianolic acid G were isolated from *Salvia miltiorrhiza* in the authors’ laboratory. Their chemical structures (Supplementary Figure S1) were unambiguously identified by comparison of the NMR and MS spectra data with reported literatures. The purity of each reference compound was determined to be higher than 98% by normalization of the peak areas detected by HPLC-UV. LPS from Escherichia coli 055:B5 was purchased from Sigma (USA). Antibody against iNOS was obtained from Cell Signaling Technology (USA); antibodies against COX-2 and β-actin were purchased from Santa Cruz Biotechnology (Santa Cruz, USA).

### Sample preparation

Cardiotonic Pill (Lot: 110419) was obtained from Tasly Pharmaceutical Co., Ltd. (China). Combinations of 10 phenolic acids (PA), 4 ginsenosides (GN), 4 tanshinones (TN) and all the 18 active compounds (BECCs) were prepared by mixing reference compounds according to their actual concentrations in CP, which have been determined in our previous study. The constituents of the combinations are detailed in Supplementary Table S1.

### Cell culture and cell viability assay

The RAW264.7 murine macrophage cell line was purchased from American Type Culture Collection (ATCC, USA) and cultured in Dulbecco’s modified Eagle’s medium containing 10% fetal bovine serum, 100 U/ml penicillin and 100 μg/ml streptomycin at 37 °C in a humidified 5% CO_2_ atmosphere. All culture reagents were purchased from Life Technologies (USA). RAW264.7 cell viability was measured using a cell counting kit-8 (CCK-8 kit, Dojido, Japan) according to the manufacturer’s instruction. Briefly, cells (5 × 10^4^ cells/well) were plated onto 96-well plates and treated with different concentrations of indicated compounds, compound combinations or CP in the presence of 1 μg/ml LPS for 24 h. CCK-8 (10 μl) was added to each well and incubated at 37 °C for 1 h followed by absorbance detection at 450 nm using a microplate reader (synergy 2, BioTek, USA).

### Nitrite measurement

RAW264.7 cells (5 × 10^4^ cells/well) were plated in 96-well plates for 12 h, and treated with different concentrations of indicated compounds or compound combinations for 1 h, followed by treatment with LPS (1 μg/ml) and incubation for an additional 24 h. The nitrite accumulation in the supernatant was determined by Griess assay^51^. Briefly, 100 μl of cell-free culture media was reacted with 50 μl of 1% sulfanilamide in 5% phosphoric acid and 50 μl of 0.1% N-(1-naphthyl)ethylenediamine in water. The absorbance was measured at 550 nm.

### PGE_2_ and IL-6 measurement

RAW264.7 cells (3 × 10^5^ cells/well) were plated in 24-well plates for 12 h, and treated with different concentrations of indicated compounds or compound combinations for 1 h, followed by treatment with LPS (1 μg/ml) and incubation for an additional 24 h. The concentration of PGE_2_ (BD Biosciences, USA) and IL-6 (Cusabio Biotech, China) in cell culture supernatants was determined using commercial available ELISA kit according to the manufacturers’ instructions.

### Evaluation of combination effect between PA and TN by combination index (CI)

The investigation of interaction in combinations involves establishing dose-effect curves for single compounds alone and for multiple combinations of agents. The ability for suppressing inflammatory mediator levels of single compounds, combinations of 10 phenolic acids (PA), combinations of 4 tanshinones (TN), combinations of PA and TN were tested at various concentrations. The suppressing ability is calculated from the following equation:


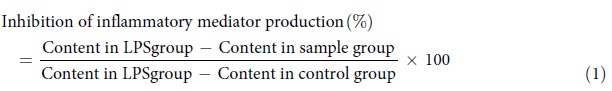


The combination effect of drugs can be characterized by the combination index (CI), which is defined as:


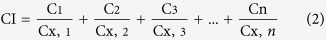


Where C_x,n_ is the concentration of Compound_n_ alone that inhibits x%. C_n_ is the concentration of Compound_n_ in combination C_1_ + C_2_ + C_3_ +…+ C_n_, which inhibits x%.

The interaction between PA and TN can be characterized by the following equation:


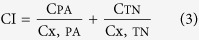


When the CI is equal to, less than or greater than 1, the combination effect is additive, synergistic or antagonistic, respectively. In this study, CI of 1 ± 0.1 is considered additive, CI value below 0.9 is considered synergistic and CI value above 1.1 is considered antagonistic. Additive effect is defined as the combined effect predicted by the mass-action law principle, synergism is as the production of a greater than expected additive effect, and antagonism is as the production of smaller than expected additive effect^45^,^46^. Inhibitory concentration 50 (IC_50_) and CI values were calculated using CompuSyn software (ComboSyn Inc., USA).

### Calculation of activity contribution from active compounds with additive effect

The activity contribution from active compounds with additive effect can be characterized by the activity contribution index (ACI_x_), which is defined as:


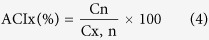


Where C_x,n_ is the concentration of Compound_n_ alone that inhibits x%. C_n_ is the concentration of Compound_n_ in combination C_1_ + C_2_ + C_3_ +…+ C_n_, which inhibits x%.

### Quantitative real-time reverse transcription-polymerase chain reaction (qRT-PCR)

RAW264.7 cells (1.5 × 10^6^ cells/well) were plated in six-well plates, incubated for 12 h, and treated with testing samples for 1 h, followed by treatment with LPS (1 μg/ml) and incubation for an additional 6 h (to quantify the mRNA of iNOS, IL-6 and IL-1β) or 12 h (to quantify the mRNA of COX-2). Total RNA was extracted using the RNAprep pure Kit (TIANGEN, China) according to the manufacturer’s instructions and stored at −80 °C until used. From each sample, 1 mg of RNA was reverse transcribed to cDNA using the PrimeScript RT reagent kit (TaKaRa, China). The primer sequences were shown in Supplementary Table S5 and were synthesized by GENEWIZ Inc. Real-time PCR analysis was performed using a LightCycler 96 System (Roche Diagnostics, Germany) with FastStart Essential DNA Green Master (Roche Diagnostics, Germany). The real time-PCR was performed with the following conditions: 95 °C for 10 min, followed by 45 cycles of 95 °C for 10 s, 60 °C for 10 s and 72 °C for 10 s. To confirm specificity of amplification, a melting curve analysis was performed at the end of each PCR program. GAPDH was used as the housekeeping gene for normalization.

### Western blotting

RAW264.7 cells (1.5 × 10^6^ cells/well) were plated in six-well plates for 12 h, and treated with testing samples for 1 h, followed by treatment with LPS (1 μg/ml) and incubation for an additional 24 h. The cells were washed twice with phosphate-buffered saline (PBS) and lysed using RAPI lysis buffer (Beyotime, China). The protein concentrations were determined using bicinchoninic acid (BCA) Protein Assay Kit (Beyotime, China). Protein samples (30 μg) from each lysate were separated on 10% SDS-polyacrylamide gel and transferred to nitrocellulose membranes (Millipore, USA) and blocked with 5% non-fat milk in Tris-buffered saline containing 0.1% Tween-20 (TBST) at room temperature for 2 h. The membranes were then incubated at 4 °C overnight with primary antibodies against iNOS, COX-2 and β-Actin. Secondary antibodies were HRP-conjugated goat anti-rabbit or anti-mouse antibodies (Beyotime, China). The blots were developed with ECL Western Blotting Substrate (Thermo Fisher Scientific) and analyzed by scanning densitometry using a Tanon Image System (Tanon, China). β-Actin was used as an interval control.

### Statistical analysis

Statistical analysis was performed with GraphPad Prism 6.0 software (GraphPad, USA). The results are expressed as the mean ± standard deviation of individual values from at least three independent experiments. Data were compared by one-way ANOVA followed by a Dunnett post-hoc test. The differences were considered statistically significant when p < 0.05.

## Additional Information

**How to cite this article**: Long, F. *et al*. A strategy for the identification of combinatorial bioactive compounds contributing to the holistic effect of herbal medicines. *Sci. Rep*. **5**, 12361; doi: 10.1038/srep12361 (2015).

## Supplementary Material

Supplementary Information

## Figures and Tables

**Figure 1:**
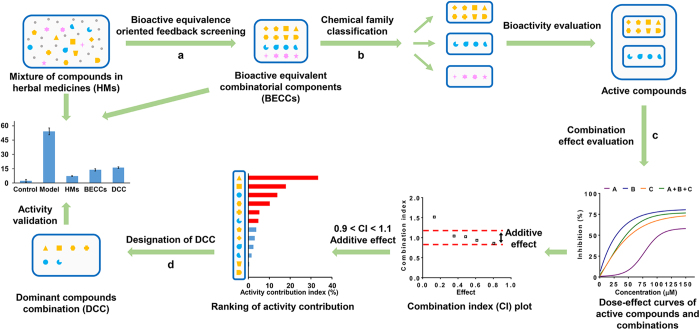
The strategy used to screen combinatorial compounds that take effect via an additive mode in HMs. (**a**) the identification of BECCs via a bioactive equivalence oriented feedback screening approach; (**b**) screening active compounds based on chemical family classification; (**c**) determining the pharmacological activity of individual compounds and their interactive mode; (**d**) designation of DCC based on activity contribution index and activity validation of DCC. BECCs: bioactive equivalent combinatorial components; DCC: dominant compounds combination.

**Table 1:** IC_50_ and activity contribution of 10 phenolic acids and 4 tanshinones.

No.	Compound	NO			IL-6		
		C (μM)	C_50_ (μM)	ACI_50_ (%)	C (μM)	C_50_ (μM)	ACI_50_ (%)
P1	Danshensu	22.21	123.2 ± 4.2	18.0	14.81	186.7 ± 9.2	7.4
P2	Protocatechuic aldehyde	9.18	27.5 ± 2.0	33.4	6.12	72.1 ± 3.6	8.5
P3	Salvianolic acid D	2.94	>200	1.5	1.96	42.8 ± 2.2	4.6
P4	Salvianolic acid G	2.74	116.2 ± 9.1	2.4	1.83	>200	0.9
P5	Isolithospermic acid A	2.68	51.0 ± 4.0	5.3	1.79	4.8 ± 0.4	37.1
P6	Salvianolic acid A	2.43	23.7 ± 1.5	10.2	1.62	6.6 ± 0.5	24.5
P7	Isolithospermic acid B	1.92	51.5 ± 3.6	3.7	1.28	20.0 ± 1.6	6.4
P8	Rosmarinic acid	1.44	96.1 ± 7.2	1.5	0.96	36.9 ± 0.9	2.6
P9	Salvianolic acid B	0.86	59.7 ± 3.9	1.4	0.57	8.8 ± 0.6	6.5
P10	Lithospermic acid	0.65	146.2 ± 10.8	0.4	0.43	26.3 ± 1.2	1.6
T1	Tanshinone I	0.44	9.2 ± 0.4	4.8	0.29	16.0 ± 1.2	1.8
T2	Dihydrotanshinone I	0.39	2.8 ± 0.2	13.9	0.26	4.8 ± 0.3	5.4
T3	Tanshinone IIA	0.30	17.5 ± 1.3	1.7	0.20	26.2 ± 2.1	0.8
T4	Cryptotanshinone	0.24	8.0 ± 0.3	3.0	0.16	15.0 ± 0.7	1.1

ACI_50_ (%) = (C_n_ / C_50,n_) × 100. ACI_50_: activity contribution index at the median effect, is defined as activity contributions of single compounds in combination of PA and TN that inhibits 50%. C_n_: concentration of Compound_n_ in combination of PA and TN that inhibits 50%. C_50,n_: also known as IC_50_, inhibitory concentration 50. The combination of PA and TN inhibited 50% of NO production at a corresponding concentration in 0.15 mg/ml CP, inhibited 50% of IL-6 production at a corresponding concentration in 0.10 mg/ml CP.
