# Supplementary material for: A strategy for the identification of combinatorial bioactive compounds contributing to the holistic effect of herbal medicines
Source: Sci Rep. 2015 Jul 22;5:12361. doi: 10.1038/srep12361 (PMC4510521; doi:10.1038/srep12361)
Supplement: Supplementary Information [file srep12361-s1.pdf]

Supplementary information

A strategy for the identification of combinatorial bioactive compounds contributing to the holistic effect of herbal medicines

Fang Long [1], Hua Yang [1], Yanmin Xu, Haiping Hao * & Ping Li *

State Key Laboratory of Natural Medicines, China Pharmaceutical University, Nanjing 210009, China

Correspondence and requests for materials should be addressed to P.L. (liping2004@126.com) or H.H. (hhp_770505@hotmail.com)

[1] These authors contributed equally to this work.

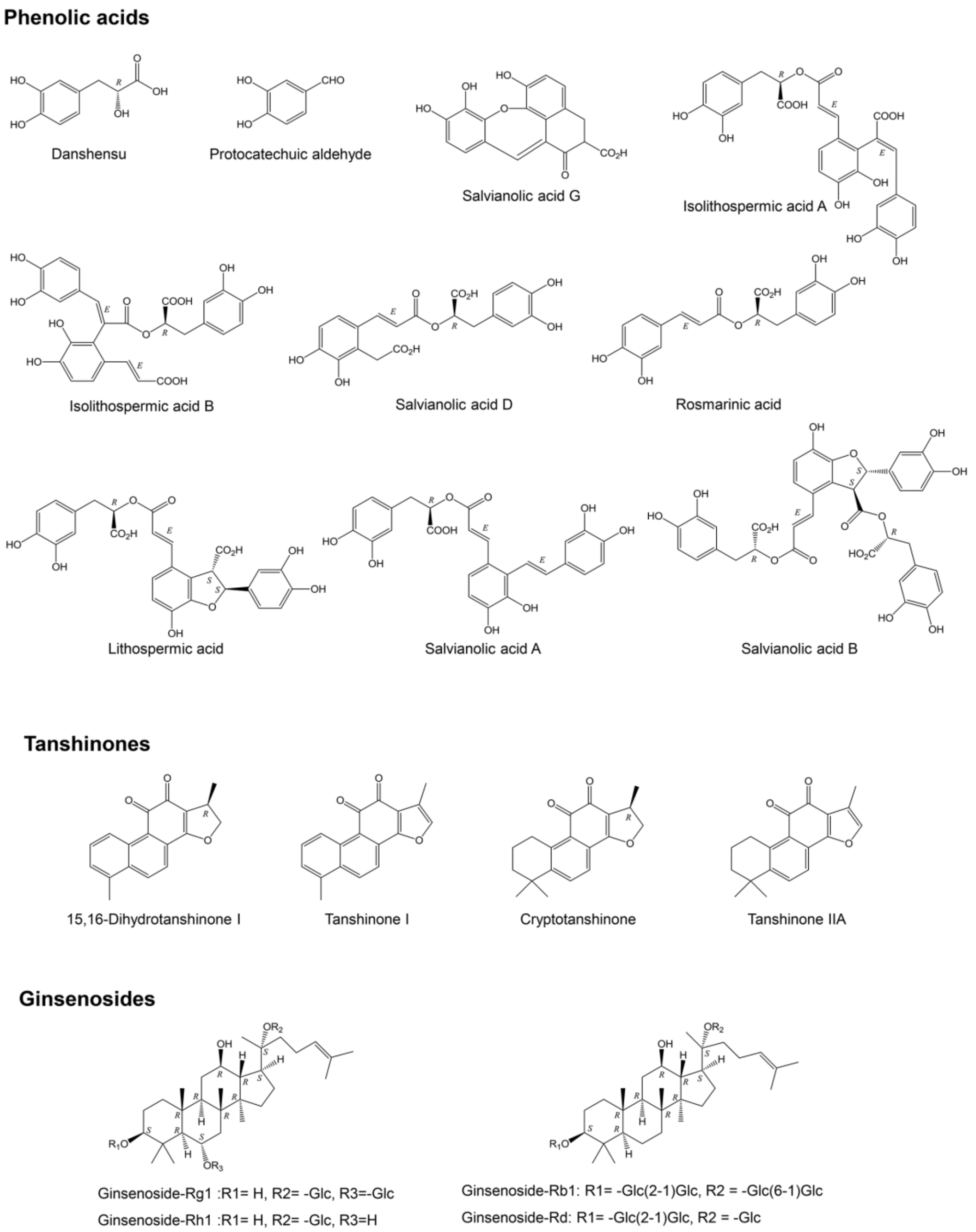

Figure S1 | Chemical structures of 18 compounds in BECCs of Cardiotonic Pill.

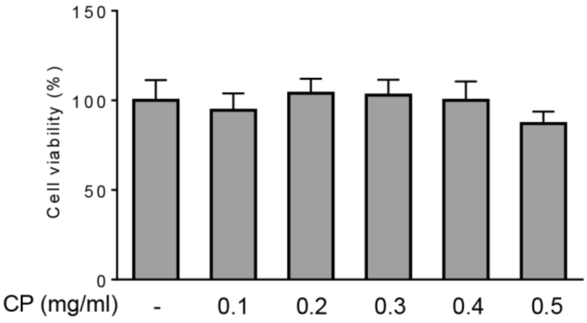

Figure S2 | Effects of Cardiotonic Pill (CP) on the cell viability of RAW264.7 macrophages. RAW264.7 cells were treated with 0.1, 0.2, 0.3, 0.4 and 0.5 mg/ml of CP in the presence of 1 μg/ml LPS for 24 h. Cell viability was measured using CCK-8 assay and expressed as mean ± SD of three independent experiments.

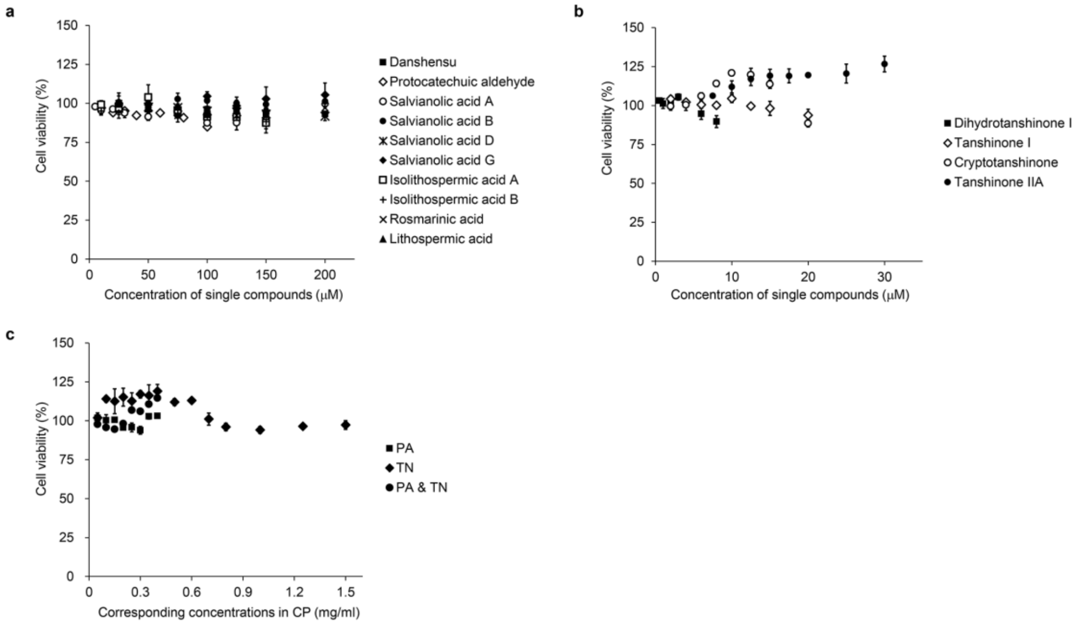

Figure S3 | Effects of single compounds and compound combinations on the cell viability of RAW264.7 macrophages. RAW264.7 macrophage cells were treated with different concentration of tested samples in the presence of 1 μg/ml LPS for 24 h. (a) 10 phenolic acids; (b) 4 tanshinones; (c) the combination of 10 phenolic acids (PA), the combination of 4 tanshinones (TN), and the combination of PA and TN (PA & TN). Cell viability was measured using CCK-8 assay and expressed as mean ± SD of three independent experiments.

Table S1 | Concentrations of 18 compounds in Cardiotonic Pill.

Group	No.	Compound	Molecular weight	Concentration in 1.0 mg/ml CP (μg/ml)	Concentration in 0.1 mg/ml CP (μM)	Concentration in 0.2 mg/ml CP (μM)	Concentration in 0.3 mg/ml CP (μM)	Concentration in 0.4 mg/ml CP (μM)
Phenolic acids (PA)	P1	Danshensu	198.17	29.34	14.8	29.61	44.41	59.21
	P2	Protocatechuic aldehyde	138.12	8.46	6.12	12.25	18.37	24.49
	P3	Salvianolic acid D	418.35	8.20	1.96	3.92	5.88	7.84
	P4	Salvianolic acid G	340.28	6.21	1.83	3.66	5.48	7.31
	P5	Isolithospermic acid A	538.46	9.61	1.79	3.57	5.36	7.14
	P6	Salvianolic acid A	494.45	8.00	1.62	3.24	4.85	6.47
	P7	Isolithospermic acid B	538.46	6.90	1.28	2.57	3.85	5.13
	P8	Rosmarinic acid	360.31	3.46	0.96	1.92	2.88	3.84
	P9	Salvianolic acid B	718.61	4.14	0.58	1.16	1.73	2.31
	P10	Lithospermic acid	538.46	17.43	0.44	0.87	1.31	1.74
Tanshinones (TN)	T1	Tanshinone I	276.29	0.47	0.30	0.59	0.89	1.18
	T2	Dihydrotanshinone I	278.3	0.81	0.26	0.52	0.78	1.04
	T3	Tanshinone IIA	294.34	0.72	0.20	0.4	0.59	0.79
	T4	Cryptotanshinone	296.36	0.58	0.16	0.32	0.47	0.63
Ginsenosides (GN)	G1	Ginsenoside Rg1	801.01	22.42	2.18	4.35	6.53	8.7
	G2	Ginsenoside Rb1	1109.29	7.03	2.02	4.04	6.06	8.08
	G3	Ginsenoside Rh1	638.87	2.56	1.10	2.20	3.30	4.40
	G4	Ginsenoside Rd	946.15	0.72	0.27	0.54	0.81	1.08

CP: Cardiotonic Pill.

Table S2 | Combination index (CI) calculation of combinations of PA and TN.

Inflammatory indicator	Effect x (%)	CI	C_{PA} * (mg/ml)	C_{TN} * (mg/ml)	$C_{x,PA}$ *,** (mg/ml)	$C_{x,TN}$ *,** (mg/ml)
NO	35.5	1.04	0.10	0.10	0.12	0.51
	48.7	1.03	0.15	0.15	0.19	0.60
	61.4	0.93	0.20	0.20	0.31	0.69
	68.9	0.92	0.25	0.25	0.42	0.75
	80.6	0.86	0.35	0.35	0.75	0.90
	82.2	0.92	0.40	0.40	0.82	0.92
IL-6	47.7	1.05	0.10	0.10	0.11	0.90
	67.8	0.93	0.15	0.15	0.18	1.37
	76.5	0.94	0.20	0.20	0.24	1.70
	82.0	0.95	0.25	0.25	0.30	2.02
	85.5	0.97	0.30	0.30	0.36	2.30
	90.9	0.82	0.35	0.35	0.50	3.00

* Corresponding concentration in CP. ** Calculated from actual experimental points.

CI: combination index; PA: the combinations of 10 phenolic acids; TN: the combination of 4 tanshinones. C_{PA}: the concentration of PA in combination PA + TN, which inhibits x%; C_{TN}: the concentration of TN in combination PA + TN, which inhibits x%; $C_{x,PA}$: the concentration of PA alone that inhibits x%; $C_{x,TN}$: the concentration of TN alone that inhibits x%.

$$CI = \frac{C_{PA}}{C_{x,\,PA}} + \frac{C_{TN}}{C_{x,\,TN}}$$

Table S3 | Combination index (CI) calculation of combinations of 10 phenolic acids (PA).

Inflammatory indicator	Effect x (%)	CI	C_n (μM)										$C_{x,n}$ (μM) *									
			P1	P2	P3	P4	P5	P6	P7	P8	P9	P10	P1	P2	P3	P4	P5	P6	P7	P8	P9	P10
NO	16.6	1.09	7.4	3.1	1.0	0.9	0.9	0.8	0.6	0.5	0.3	0.2	65.8	5.2	134.0	42.7	9.5	5.0	13.6	60.1	8.3	29.6
	34.8	0.89	14.8	6.1	2.0	1.8	1.8	1.6	1.3	1.0	0.6	0.4	96.5	14.4	172.8	78.7	26.5	12.9	30.7	80.1	27.7	78.5
	48.2	1.10	29.6	12.2	3.9	3.7	3.6	3.2	2.6	1.9	1.2	0.9	119.9	25.5	199.5	111.2	47.4	22.1	48.5	94.2	54.8	136.2
	54.3	1.13	37.0	15.3	4.9	4.6	4.5	4.0	3.2	2.4	1.4	1.1	131.8	32.8	212.4	129.3	61.0	28.0	59.3	101.1	73.7	173.3
	61.8	1.05	44.4	18.4	5.9	5.5	5.4	4.9	3.8	2.9	1.7	1.3	148.7	45.2	230.2	156.8	84.4	37.8	76.6	110.6	107.7	235.7
	67.5	1.01	51.8	21.4	6.9	6.4	6.2	5.7	4.5	3.4	2.0	1.5	163.8	58.5	245.4	183.0	109.4	48.1	94.0	118.9	145.9	301.4
IL-6	43.9	1.38	14.8	6.1	2.0	1.8	1.8	1.6	1.3	1.0	0.6	0.4	160.6	44.7	34.4	186.6	3.4	5.1	15.0	28.7	6.4	19.9
	63.1	1.06	22.2	9.2	2.9	2.7	2.7	2.4	1.9	1.4	0.9	0.7	313.6	143.5	48.2	326.8	7.1	8.5	26.0	44.0	12.3	33.9
	71.5	1.04	29.6	12.2	3.9	3.7	3.6	3.2	2.6	1.9	1.2	0.9	435.3	254.2	56.9	430.0	10.2	10.9	34.1	54.2	16.8	44.0
	77.3	1.02	37.0	15.3	4.9	4.6	4.5	4.0	3.2	2.4	1.4	1.1	565.5	400.9	65.0	535.3	13.7	13.4	42.2	64.0	21.7	54.2
	82.9	1.08	51.8	21.4	6.9	6.4	6.2	5.7	4.5	3.4	2.0	1.5	765.0	678.9	75.7	689.5	19.1	16.9	54.1	77.6	29.0	69.0
	90.5	0.74	59.2	24.5	7.8	7.3	7.1	6.5	5.1	3.8	2.3	1.7	1363	1859	101.5	1118	36.4	26.4	87.0	112.1	50.7	109.5

* Calculated from actual experimental points.

CI: combination index; PA: the combinations of 10 phenolic acids; P1: danshensu; P2: protocatechuic aldehyde; P3: salvianolic acid D; P4: salvianolic acid G; P5: isolithospermic acid A; P6: salvianolic acid A; P7: isolithospermic acid B; P8: rosmarinic acid; P9: salvianolic acid B; P10: lithospermic acid; C_n: the concentration of Compound$_n$ in combination $C_1 + C_2 + C_3 + \ldots + C_n$, which inhibits x%; $C_{x,n}$: the concentration of Compound$_n$ alone that inhibits x%.

$$CI = \frac{C_1}{C_{x,1}} + \frac{C_2}{C_{x,2}} + \frac{C_3}{C_{x,3}} + \ldots + \frac{C_n}{C_{x,n}}$$

Table S4 | Combination index (CI) calculation of combinations of 4 tanshinones (TN).

Inflammatory indicator	Effect x (%)	CI	C_n (μM)				$C_{x,n}$ (μM) *			
			T1	T2	T3	T4	T1	T2	T3	T4
NO	9.2	1.76	0.8	0.7	0.5	0.4	3.5	0.5	7.1	3.0
	34.2	1.26	1.6	1.4	1.1	0.9	7.0	1.8	13.6	6.1
	50.6	1.01	1.9	1.7	1.3	1.0	9.3	2.9	17.7	8.1
	61.3	0.97	2.4	2.1	1.6	1.3	11.1	3.9	21.0	9.8
	74.9	0.90	3.2	2.8	2.2	1.7	14.5	6.2	26.9	12.9
	80.3	0.94	4.0	3.6	2.7	2.2	16.6	7.7	30.4	14.8
IL-6	19.8	1.34	1.2	1.0	0.8	0.6	5.4	1.1	11.5	4.9
	29.1	1.08	1.5	1.3	1.0	0.8	7.7	1.8	14.9	7.1
	40.0	0.99	2.1	1.8	1.4	1.1	10.8	2.9	19.0	10.0
	52.4	0.93	3.0	2.6	2.0	1.6	15.3	4.7	24.6	14.3
	57.2	1.02	3.7	3.3	2.5	2.0	17.6	5.7	27.1	16.5
	58.7	1.13	4.4	3.9	3.0	2.4	18.3	6.1	27.9	17.2

* Calculated from actual experimental points.

CI: combination index; TN: the combinations of 4 tanshinones; T1: tanshinone I; T2: dihydrotanshinone I; T3: tanshinone IIA; T4: cryptotanshinone; C_n: the concentration of Compound_n in combination $C_1 + C_2 + C_3 + \ldots + C_n$, which inhibits x%; $C_{x,n}$: the concentration of Compound_n alone that inhibits x%.

$$CI = \frac{C_1}{C_{x,1}} + \frac{C_2}{C_{x,2}} + \frac{C_3}{C_{x,3}} + \ldots + \frac{C_n}{C_{x,n}}$$

Table S5 | Primer sequences for qRT-PCR.

Gene name	Accession number	Forward primer	Reverse primer
iNOS	NM_010927.3	CATGCTACTGGAGGTGGGTG	CATTGATCTCCGTGACAGCC
COX-2	NM_011198.3	CCTCTGCGATGCTCTTCC	TCACACTTATACTGGTCAAATCC
IL-1β	NM_008361.3	ACCTGCTGGTGTGTGACGTT	TCGTTGCTTGGTTCTCCTTG
IL-6	NM_031168.1	GAGGATACCACTCCCAACAGACC	AAGTGCATCATCGTTGTTCATACA
GAPDH	NM_008084.3	TTCACCACCATGGAGAAGGC	GGCATGGACTGTGGTCATGA
